# Radiation therapist led treatment of lung stereotactic ablative body radiation therapy patients in the absence of the radiation oncologist – An image matching consistency comparison study

**DOI:** 10.1016/j.tipsro.2026.100376

**Published:** 2026-01-07

**Authors:** Menglei Chao, Kylie Unicomb, Maryam Hazem, Shamira Cross, Gary Low, Roland Yeghiaian-Alvandi

**Affiliations:** a Nepean Cancer and Wellness Centre, Kingswood 2747, Australia; b Nepean Clinical School, University of Sydney, Australia; c Research Directorate, Nepean Hospital, Nepean Blue Mountain Local Health District, Derby St, Kingswood, NSW 2750, Australia; d Sydney Medical School, Faculty of Medicine and Health, University of Sydney, Australia

**Keywords:** Lung SABR, Pre-treatment CBCT, SABR treatment credentialing, Image registration

## Abstract

**Introduction:**

The aim of this study was to evaluate the consistency of day 1 cone-beam computed tomography (CBCT) image registration in lung stereotactic ablative body radiation therapy (SABR) among radiation oncologists (RO), radiation therapists (RTT) and paired RTTs, negating the need for RO to attend day 1 lung SABR treatment.

**Methods:**

Day 1 pre-treatment CBCT data sets were restored for ten lung SABR patients for offline image registration. All participants (2 ROs, 27 RTTs and 5 RTT pairs) performed image matches on three different days. The deviations between each offline match and day 1 reference data were analysed to assess the consistency of image registration among the three study groups. Other variables such as patient respiratory motion management method, RTT SABR experience, and time spent on image registration were evaluated.

**Results:**

All three study groups showed excellent consistency in CBCT matching results when compared to reference data. Average differences in all translational and rotational directions for each group were ΔTx 0.02 cm, ΔTy 0.04 cm, ΔTz 0.02 cm, ΔRx 0.43°, ΔRy 0.43°, ΔRz 0.47° for RT, ΔTx 0.02 cm, ΔTy 0.04 cm, ΔTz 0.04 cm, ΔRx 0.54°, ΔRy 0.66°, ΔRz 0.48° for RO and ΔTx 0.02 cm, ΔTy 0.06 cm, ΔTz 0.04 cm, ΔRx 0.39°, ΔRy 0.47°, ΔRz 0.49° for RTT pair. All deviations were within the study tolerance.

**Conclusions:**

Following appropriate education and training, lung SABR credentialed RTTs demonstrated the ability to perform high quality and consistent day1 pre-treatment CBCT matching potentially negating the need for routine approval from ROs.

## Highlights

• Excellent Consistency in Image Matching Across Observer Groups.
• Respiratory motion management strategy significantly affected image consistency.
• Effective training program equips RTs with the necessary skills for lung SABR CBCT registration.

## Background

Stereotactic Ablative Body Radiation Therapy (SABR) is a treatment technique used to treat early-stage non-small cell lung cancer (NSCLC) and lung metastasis. This SABR treatment technique aims to ablate the cancer with large doses of radiation with a high degree of geometric accuracy, and with creation of steep dose fall off gradients to spare surrounding organs at risk (OARs), including healthy lung tissue [1]. Lung SABR is considered the standard of care in peripheral NSCLC in patients that are not surgical candidates [2], [3]. To plan and deliver Lung SABR treatments accurately, respiratory motion management strategies are needed to reduce respiratory motion, the motion artefacts during image acquisition, visualise the lesion and ensure respiration motion does not exceed what was intended when the Radiation Oncologist (RO) contoured the Internal Target Volume (ITV) or the Gross Target Volume (GTV). Deep Inspiration Breath Hold (DIBH) and Free Breathing (FB) with ITV delineation are 2 respiratory motion management strategies used in this study.

Lung SABR is a technical and precise treatment technique, which requires teamwork and collaboration from a multidisciplinary team (MDT). Members of this team include the RO, credentialled Radiation Therapists (RTT) as well as Radiation Oncology Medical Physicists (ROMP) SABR specialists [4]. Each discipline has input in every part of the patient’s SABR journey, from selecting the most appropriate respiratory motion management strategy, image quality assessment, multi-modality image registration, target delineation, plan creation, quality checks, and treatment delivery. Since implementation of SABR at our centre the standard of care is for a representative of each discipline to attend all day 1 Lung SABR treatments to assess and approve the online Image Guided Radiation Therapy (IGRT) process. The RTTs perform the match, RO to approve it and ROMP to provide technical oversight and support as needed for these complex treatments to ensure accurate and efficient delivery of the service. Minimum-standards guidance, such as the Guidelines For Safe Practice of Stereotactic Body (Ablative) Radiation Therapy [7] model, emphasises strong governance, clear protocols, and multidisciplinary oversight to ensure safe SABR delivery.

Cone-beam computed tomography (CBCT) imaging can provide sufficient information for tumour localisation as proven in the literature [5], [6]. According to the guidelines for safe practice of stereotactic body (ablative) radiation therapy written by Foote et al [7], the standard of care when SABR is first implemented recommends representatives from RO, RTT and ROMP to attend the first treatment. This document goes on to indicate that once the SABR service has been established, the roles and responsibilities can be re-visited based on staff competence and number of patients treated. A study undertaken in Australia found that RTTs alone produced more consistent and accurate results when compared to the match used clinically to treat the 40 patients used in this study [8]. A Canadian study by Kim et al. [9] demonstrated high interobserver reliability between RTTs and ROs during lung SABR image matching. The authors concluded that systematic RO approval may not be required, as no clinically significant discrepancies were observed between the two professional groups. Another study in the United Kingdom also proved therapeutic radiographers can provide comparable CBCT verification matches to clinical oncologists’gold standard’ [10].

The aim of this study was to evaluate the variability of IGRT matching between RTTs and ROs. If the data analysis demonstrates consistency between RTT and RO matches, it could potentially support the removal of RO attendance for Lung SABR treatments under certain conditions.

## Method and Materials

Twenty-seven RTTs, two ROs, and five pairs of RTTs were recruited to perform offline CBCT image registration on ten consecutively treated lung SABR patients between 2020 and 2021, using an Elekta VersaHD linear accelerator (Elekta, Crawley, England). Selecting consecutively treated patients shows expected variation in patient demographics, disease staging, lesion location, PTV size and OARs. These considerations have not impacted the use of motion management or IGRT matching workflows. Lung SABR patients in our department were treated either using FB or DIBH. For FB patients, 4D CT and 4D CBCT were used to assess free breathing motion. All DIBH patients used a spirometer-based valve system, the Active Breathing Coordinator (ABC), to achieve breath hold. Patients in this study were immobilised in an Elekta BodyFix T shaped vacbag with indexed knee supports enabling 3 points of fixation. Participation in the study was voluntary, and invitations were sent to all twenty-seven credentialled RTTs. The limit of five RTT skill-mixed pair matches enabled the continuation of clinical services with minimal impact while still reflecting the diversity of practice in a clinical setting. Participating RTTs were credentialed for lung SABR treatment following a comprehensive departmental training program. Both ROs are specialized in lung SABR treatment. The RTT pairs were randomly selected from the credentialed RTT pool to simulate a real-life clinical scenario where radiotherapy is delivered by RTT pairs.

The department training program incorporates 5 key elements: theoretical SABR foundation learning, offline CBCT review, observational learning, live CBCT performance and continuous learning. A comprehensive written assessment is applied to evaluate the trainee's understanding of fundamental SABR principles after theory learning. Subsequently, trainees analyse at least 4 lung SABR cases offline with different target locations and motion management strategies with the assessor. Following the offline session, trainees observes and performs several online CBCT matches with the supervision of an assessor before deemed competent. This stringent training program equips RTTs to assess and interpret IGRT matches and the dosimetric impact as RTTs in Australia are also responsible for planning radiation therapy treatments.

Day 1 pre-treatment CBCT and planning CT data sets were restored to image registration software XVI 5.04 for offline image matching. Each participant. RTT, RO and RTT pairs performed 10 CBCT registration tasks on three different days with a minimum interval of 24 h between sessions. In total, there are 30 CBCT matches per participant / pair performed. While this design utilised 10 datasets the repeated sessions resulted in a total of 1019 CBCT correction and 6114 directional data points collected for analysis. Automatic dual registrations were performed with rigid bone alignment and soft tissue match based on a mask created 0.5 cm from ITV. Participants reviewed the automatic registration results and manually adjusted the target position if necessary following standard department lung SABR IGRT guidelines. Translational (Tx = left/right, Ty = sup/inf, Tz = ant/post) and rotational (Rx = pitch, Ry = roll, Rz = yaw) corrections were then recorded as final matching results.

For this study, Day 1 treatment images were chosen as a clinically sensitive point of lung SABR treatment, with matching performed by the RTTs and approved by the responsible RO for treatment. This match establishes the baseline for all subsequent treatments considering patient-specific IGRT considerations and is used as the gold standard for comparison. By using this clinically validated dataset as gold standard, we ensured both consistency and relevance when evaluating the accuracy of the offline registrations performed by study participants. Each offline matching result was compared to the gold standard, to determine the absolute differences between these 2 sets of data (ΔT and ΔR). Consistency in this study was defined using our established clinical tolerance for lung SABR imaging: 2 mm for translation and 2 degrees for rotation. Clinical IGRT tolerance was established based on ROMP led internal modelling and patient specific calculations considering dosimetric impact of shifts of 2 mm and 2 degrees accounting for patient movement, OAR tolerance and target coverage. All patients included in this study underwent this assessment and shifts within these tolerances did not produce clinically meaningful changes to dosimetry. RTT participants (both individual group and RTT pair group) were also instructed to record their time spent on each image match.

Ethical approval for this study was obtained from the Human Research Ethics Committees (HREC) (2022/ETH00783). Data collection was conducted and managed using the Research Electronic Data Capture (RedCap) system. Statistical analysis included the Intraclass Correlation Coefficient (ICC), Bland-Altman analysis and independent samples t-tests. Statistical significance was defined as p < 0.05.

## Results

Table 1 summarises the general and radiation treatment characteristics of the ten recruited patients. Among all recruited patients, there were five females and five males aged 68 to 86 years old. The lesions were located in the upper lobe (n = 6) and lower lobe (n = 4) of the lungs, with seven lesions in the right lung and three in the left lung. Six patients were treated using FB with 4D CBCT for motion management and four patients were treated under DIBH. The PTV sizes ranged from 4.91 cm^3^ to 80.06 cm^3^. A total of 1019 sets of CBCT data were collected, encompassing 6114 data points (excluding one invalid dataset due to user error).

**Table 1:** Patient general and radiation treatment characteristics.

Pt #	Age	Gender	Planning target volume location	Planning target volume (cc)	Dose (Gy/F)	Respiratory motion management
1	70	F	RLL	13.08	48/4	DIBH
2	86	F	RUL	21.55	48/4	DIBH
3	76	F	RLL	80.06	50/5	FB
4	73	M	RLL	23.36	48/4	DIBH
5	76	M	RUL	4.91	48/4	FB
6	80	M	RUL	28.38	50/5	FB
7	82	M	LLL	14.58	48/4	FB
8	68	F	LUL	10.82	48/4	FB
9	74	M	RUL	31.89	24/3	FB
10	77	F	LUL	5.41	48/4	DIBH

### RTT vs RO vs RTT pair

The averages absolute translational and rotational correction for each study group were Tx 0.21 cm, Ty 0.1 cm, Tz −0.14 cm and Rx 0.94°, Ry −0.61°, Rz 0.05° for RO group, Tx 0.2 cm, Ty 0.1 cm, Tz −0.14 cm and Rx 0.96°, Ry −0.4°, Rz −0.03° for RTT group, and Tx 0.2 cm, Ty 0.12 cm, Tz −0.14 cm and Rx 0.94°, Ry −0.4°, Rz −0.03° for RTT pair group respectively. Results from each group were compared to the gold standard (Tx 0.2 cm, Ty 0.08 cm, Tz −0.14 cm, Rx 0.92°, Ry −0.53°, Rz 0.03°) to obtain the differences (ΔT and ΔR) (see Table 2). Based on reported mean differences and standard deviation (SD), all 3 groups results were well within the clinical tolerance for both translational and rotational corrections.

**Table 2:** Mean differences of translational and rotational corrections and intraclass correlation coefficient (ICC) analysis for RO, RTT and RTT Pair groups.

Group	ΔTx (L/R)	ΔTy (S/I)	ΔTz (A/P)	ΔRx (Pitch)	ΔRy (Roll)	ΔRz (Yaw)	Intraclass Correlation	95 % Confidence Interval	
	Mean (SD) (cm)	Mean (SD) (cm)	Mean (SD) (cm)	Mean (SD) (°)	Mean (SD) (°)	Mean (SD) (°)		Lower Bound	Upper Bound
RTT	0.02 (0.03)	0.04 (0.15)	0.02 (0.05)	0.43 (0.48)	0.43 (0.50)	0.47 (0.53)	0.99	0.99	0.99
RO	0.02 (0.04)	0.04 (0.09)	0.04 (0.06)	0.54 (0.60)	0.66 (0.72)	0.48 (0.55)	0.99	0.99	0.99
RTT Pair	0.02 (0.03)	0.06 (0.15)	0.04 (0.12)	0.39 (0.43)	0.47 (0.61)	0.49 (0.60)	0.99	0.99	1

Intraclass correlation coefficient (ICC) analysis was performed to assess the degree of consistency among RO, RTT and RTT pair when comparing to gold standards. Based on Cicchetti’s often quoted guidelines for interpretation [11], ICC measure was defined as excellent consistency when fell between 0.75 and 1. All 3 study groups show excellent consistency by using ICC method (see Table 2).

**Fig. 1:**
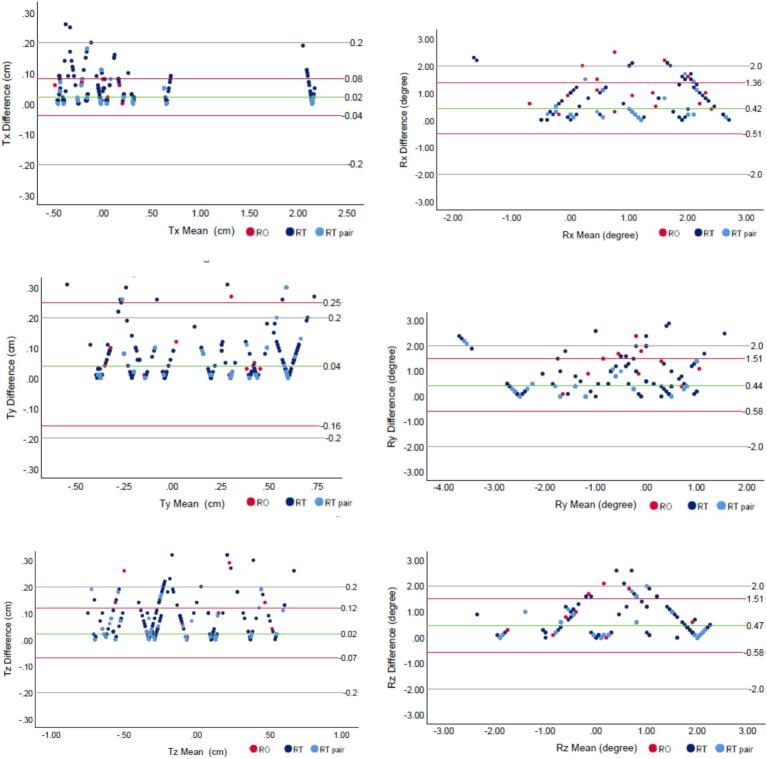
Bland-Altman plots of translational and rotational corrections.

Individual offline image matching discrepancies were visualised using Bland-Altman plots in all 6 directions (Fig. 1). Red dots represented RO group and dark blue dots for RTT group and light blue dots for RTT pair group respectively. The green line in each plot signified the mean of difference in each direction with 2 red lines representing the 95 % confidence interval above and below the mean. Additionally, the study tolerance of 2 mm for translations and 2° for rotations was depicted by 2 solid grey lines on each graph. The majority of data points fell within the defined imaging tolerance, although some scattered points were observed beyond the tolerance limits. Notably, the sup/inf direction (Ty) exhibited greater variation compared to other translational corrections, and the roll direction (Rz) showed slightly more variations than any other rotational corrections.

### FB *vs* DIBH

Differences in CBCT matching across sup/inf, lt/rt, ant/post directions based on ventilation methods were further analysed. Table 3 summarizes the mean and SD of differences for both translational (ΔT) and rotational (ΔR) discrepancies. A total of 408 and 611 sets of CBCT matching results were collected for the DIBH and FB groups, respectively. While the mean results from both breathing groups remained within the established study tolerance, independent-samples *t*-test revealed significant differences in sup/inf direction (ΔTy p < 0.01), ant/post direction (ΔTz p = 0.01) and all rotational corrections (ΔR p < 0.01) (Table 3). Furthermore, an evaluation of the number of CBCT matches exceeding the study tolerance demonstrated a higher proportion within the FB group compared to the DIBH group. Specifically, 87 out of 3666 (2.37 %) data points in FB group and 15 out of 2448 (0.61 %) data points in DIBH group exceeded the established thresholds.

**Table 3:** Mean and SD of translational and rotational differences in FB and DIBH group and independent-samples *t*-test for different breathing methods.

Breathing Method		N	ΔTX (cm)	ΔTy (cm)	ΔTz (cm)	ΔRx (°)	ΔRy (°)	ΔRz (°)
DIBH	Mean (SD)	408	0.02 (0.02)	0.03 (0.03)	0.02 (0.08)	0.22 (0.26)	0.24 (0.45)	0.37 (0.50)
FB	Mean (SD)	611	0.02 (0.04)	0.06 (0.19)	0.03 (0.06)	0.56 (0.54)	0.59 (0.55)	0.54 (0.56)
Sig. (2 tailed)			0.14	<0.00	0.01	<0.00	<0.00	<0.00
95 % Confidence interval	Lower		−0.00	0.01	0.00	0.29	0.31	0.11
	Upper		0.01	0.04	0.01	0.40	0.43	0.24

### User SABR experience

RTT User’s SABR experience was categorized into three subgroups based on the number of years of experience treating lung cancer with SABR: < 3 years (n = 9), 3–5 years (n = 4), and > 5 years (n = 14).

A total of 270, 120, and 419 sets of CBCT data were analysed within the < 3 years, 3–5 years, and > 5 years groups, respectively. ICC analysis was conducted on all ΔT and ΔR directions, demonstrating excellent consistency across all three groups when using day 1 CBCT results as the reference measurements (Table 4).

**Table 4:** ICC analysis for RTTs with different years of Lung SABR experience.

ICC	N	ΔTx	ΔTy	ΔTz	ΔRx	ΔRy	ΔRz	CBCT no.
<3 years	9	0.99	0.95	0.99	0.89	0.92	0.88	270
3–5 years	4	0.99	0.99	0.99	0.91	0.91	0.89	120
>5 years	14	0.99	0.99	0.99	0.91	0.93	0.87	419

### Time

Another variable investigated in this study was the time spent by RTTs on offline CBCT image matching. Table 5 presents the mean and SD of time taken for both individual RTTs and RTT pairs to complete the matching process. The analysis revealed that, on average, RTT pairs spent less time performing CBCT matches compared to individual RTTs. This difference was confirmed by an independent-samples *t*-test, demonstrating statistical significance (p < 0.01).

**Table 5:** Mean and SD of time taken to complete offline image matching.

	Match Details	N	Mean (SD)	Sig. (2 tailed)	95 % Confidence Interval	
					Lower	Upper
Time (seconds)	RTT	799	100.45 (66.46)	<0.01	35.29	57.06
	RTT Pair	149	54.28 (29.73)			

## Discussion

Our centre has treated lung SABR for over 10 years, establishing it as a well-developed technique for early-stage NSCLC and metastatic lung lesions. All RTTs complete a mandatory lung SABR IGRT training program to gain lung SABR treatment credentials. Patients undergo either FB or DIBH for respiratory motion management during SABR treatment. At our centre current practice for RO approval of Day 1 images matches incorporates clinical judgement for patient specific criteria accounting for lesion motion, areas of significance, previous treatment and anatomical changes. This study aims to provide departmental evidence supporting the removal of ROs from the day 1 attendance routine for CBCT image matching, prior to treatment delivery. This ensures consistent decision making to deliver an accurate SABR service delivery to our patients and is of note that should the match be of concern the treating RO will be notified to attend to provide guidance case by case.

Unlike previous studies [8], [9], this investigation included all credentialed RTTs from our centre, alongside two lung cancer specialists who routinely perform online IGRT assessment during day 1 treatment. Participants represented diverse SABR IGRT experience levels, reflecting a realistic clinical scenario. Additionally, we introduced a third observer group (“RTT pair”) consisting of two randomly selected RTTs, simulating real-world team collaboration during online CBCT review. Furthermore, the study investigated potential factors influencing IGRT consistency, such as breathing methods and RTT SABR experience.

Based on ICC analysis, ROs, RTTs and RTT pairs demonstrated excellent consistency in CBCT image matching compared to day 1 results approved by our current standard care team (comprising RTTs, ROs, and medical physicists (ROMPs) involved in online CBCT review). No statistically significant differences were observed in image matching results among the three groups.

Previous literature supports our findings. Sweeney et al. reported high agreement (96.1 % concordance) between RTTs and ROs in evaluating daily verification films [12]. Similarly, their study found minimal differences in image-guided target localization between ROs and RTTs for lung SABR (<1mm and < 2 mm for 4D and 3D CBCT, respectively) [13]. Baran et al. observed significant interobserver variability between ROs and ROMPs, as well as RTTs and ROMPs, when comparing lung SABR image registration. However, no significant difference was found between RO and RTT. They also identified some prediction metrics which can be used to predict interobserver variability such as target excursion and local target contrast. [14].

The image registration performance of the “RTT pair” group in this study suggests that credentialed RTTs in our department can perform consistent pre-treatment image registration without mandatory RO approval.

This study also investigated the influence of respiratory motion management strategies on image consistency. Compared to the DIBH group, the FB group exhibited significant differences in CBCT results for sup/inf, ant/post, and all rotational directions. Translational variations in the FB group could be attributed to factors such as the patient's breathing amplitude or manual adjustments due to blurry 4D CBCT images. Rotational variations might be related to the smaller field of view (FOV) in 4D CBCT, potentially excluding or partially obscuring the spine. According to our departmental lung SABR IGRT guideline, spine visualisation is used as a reference for automatic bone registration in defining rotational corrections. The limited spine visibility in 4D CBCT likely contributed to the observed rotational inconsistencies and the higher proportion of results exceeding the 2 mm/2° tolerance compared to the DIBH group.

As a department promoting SABR skill development among all RTT levels, we investigated SABR image consistency across different SABR experience groups (<3 years, 3–5 years, >5 years). ICC analysis revealed that RTTs from all experience levels produced CBCT match results with excellent consistency when compared to day 1 reference images. This finding indicates the effectiveness of our departmental SABR credentialing program in equipping RTTs with the necessary image registration and assessment skills for lung SABR treatment. Although a slightly higher number of out-of-tolerance results were observed in the < 3 years group, this number decreased with increasing experience.

An interesting observation was the difference in time spent on offline CBCT registration between individual RTTs and RTT pairs. While both groups achieved excellent consistency, working together as an RTT team significantly reduced time spent on image registration and evaluation. In this study, RTT pair group required half the time compared to individual RTTs for performing offline image matching. However, it is important to acknowledge that online CBCT matching times might differ due to factors such as patient positioning, anatomical complexity, target visibility and distractions around the treatment console area.

There are also some limitations to this study. The 10 lung SABR cases selected for this study may not fully represent the spectrum of all clinical scenarios. The difficulty level of CBCT matching is also limited by the cases we selected. Online CBCT assessment is not part of this study which means performance variation under time pressure is not evaluated in this study.

## Conclusion

This study provides evidence supporting the transition to an RTT-led lung SABR treatment model based on the high level of consistency demonstrated by RTTs. Our centre’s extensive experience in SABR over 10 years has fostered a highly skilled and experienced team capable of implementing RTT-led lung SABR treatment. By implementing RTT led lung SABR treatment, it will improve SABR treatment efficiency which means reduced treatment duration and sparing ROs from attending day 1 treatment. For centres that do not have regular RO attendance, it brings the potential to make SABR treatment accessible for patients of those areas.

## Declaration of Generative AI use

The authors declare that they used Gemini Google for grammar and spelling checks. After using this AI tool, the authors reviewed and edited the content as needed and take full responsibility for the content of the publication.

## Ethics approval

The ethics application of this study was reviewed by Nepean Blue Mountain Local Health District HREC and determined to meet the requirements of the National Standard on Ethical Conduct in Human Research (2007).

## Funding

None.

## Declaration of competing interest

The authors declare that they have no known competing financial interests or personal relationships that could have appeared to influence the work reported in this paper.
